# Modulation of Estrogen Response Element-Driven Gene Expressions and Cellular Proliferation with Polar Directions by Designer Transcription Regulators

**DOI:** 10.1371/journal.pone.0136423

**Published:** 2015-08-21

**Authors:** Mesut Muyan, Gizem Güpür, Pelin Yaşar, Gamze Ayaz, Sırma Damla User, Hasan Hüseyin Kazan, Yanfang Huang

**Affiliations:** 1 Department of Biological Sciences, Middle East Technical University, Ankara, 06800, Turkey; 2 Department of Biochemistry & Biophysics, University of Rochester, Rochester, NY, 14619, United States of America; Roswell Park Cancer Institute, UNITED STATES

## Abstract

Estrogen receptor α (ERα), as a ligand-dependent transcription factor, mediates 17β-estradiol (E2) effects. ERα is a modular protein containing a DNA binding domain (DBD) and transcription activation domains (AD) located at the amino- and carboxyl-termini. The interaction of the E2-activated ERα dimer with estrogen response elements (EREs) of genes constitutes the initial step in the ERE-dependent signaling pathway necessary for alterations of cellular features. We previously constructed monomeric transcription activators, or monotransactivators, assembled from an engineered ERE-binding module (EBM) using the ERα-DBD and constitutively active ADs from other transcription factors. Monotransactivators modulated cell proliferation by activating and repressing ERE-driven gene expressions that simulate responses observed with E2-ERα. We reasoned here that integration of potent heterologous repression domains (RDs) into EBM could generate monotransrepressors that alter ERE-bearing gene expressions and cellular proliferation in directions opposite to those observed with E2-ERα or monotransactivators. Consistent with this, monotransrepressors suppressed reporter gene expressions that emulate the ERE-dependent signaling pathway. Moreover, a model monotransrepressor regulated DNA synthesis, cell cycle progression and proliferation of recombinant adenovirus infected ER-negative cells through decreasing as well as increasing gene expressions with polar directions compared with E2-ERα or monotransactivator. Our results indicate that an ‘activator’ or a ‘repressor’ possesses both transcription activating/enhancing and repressing/decreasing abilities within a chromatin context. Offering a protein engineering platform to alter signal pathway-specific gene expressions and cell growth, our approach could also be used for the development of tools for epigenetic modifications and for clinical interventions wherein multigenic de-regulations are an issue.

## Introduction

Estrogen receptor (ER) α and β are ligand-dependent transcription factors [1,2]. ERs are distinct gene products expressed in the same as well as different tissues at varying levels [1,2],. ERs mediate the cellular effects of estrogen hormones, particularly the main circulating estrogen hormone 17β-estradiol (E2). E2 is involved in many physiological and pathophysiological processes of various tissue and organs [1,2]. Although the etiology of estrogen target tissue, particularly breast tissue, malignancies is multifactorial in which a polygenic background is modulated by the integrated effects of genetic, physiological, environmental and nutritional factors, aberrant E2 signaling is a major factor contributing to the ontogeny of malignancies [1,2].

Immediately after synthesis, ERα dimerizes and translocates primarily to the nucleus independent of E2 [3]. E2 binding leads to a conformational change in the carboxyl-terminus of ERα. This, in turn, generates binding surfaces for effective interactions with co-regulatory proteins [4,5] and enhances the stability [3] and the association with DNA of the ERα dimer [2,6]. The nuclear E2-bound ERα regulates gene transcriptions through estrogen response element (ERE)-dependent and ERE-independent pathways. EREs are permutations of the 5’-GGTCAnnnTGACC-3’ DNA palindrome, wherein ‘n’ denotes a non-specific three nucleotide spacer, located at various distances from the transcription start site [7,8]. The regulation of gene expressions through EREs by E2-ERα is referred to as the ERE-dependent signaling pathway. On the other hand, the transcriptional modulation of target genes through interaction of E2-ERα with transcription factors bound to their cognate regulatory elements on DNA denotes the ERE-independent signaling pathway [1,2]. While the ERE-independent signaling participates in the fine-tuning of cellular responses, E2-ER mediated gene expressions through the ERE-dependent signaling route are required for phenotypic changes in cell models [9,10].

ERS as other transcription factors are modular proteins [1,2]. The specific interaction of ERα with EREs is mediated through the centrally located DNA binding, or C, domain that contains two zinc-binding motifs, each of which nucleated through a zinc ion. Each C domain of the ERα dimer makes equivalent contacts with one half-site of the ERE palindrome, resulting in a rotationally symmetric structure [11]. Exploiting the intrinsic ERE binding ability of ERα, we previously showed that a monomeric ERE-binding module (EBM or CDC) can be engineered by genetically joining two DNA-binding domains (Cs) of ERα with its hinge domain (D) that contains a nuclear localization signal [12]. The integration of strong activation domains (ADs) from other transcription factors into this ERE binding module generated monotransactivators that robustly induced ERE-driven reporter gene expressions independent of E2 or dimerization [12]. Moreover, monotransactivators modulated cellular proliferation by activating as well as repressing the endogenous ERE-driven gene expressions in a manner similar to those mediated by E2-ERα [13].

We therefore envision that the genetic conjugation of repression domains (RDs) from other transcription regulators into the EBM could generate potent monotransrepressors that alter the expression of endogenous ERE-bearing genes and cellular proliferation in opposite directions to those observed with E2-ERα or monotransactivators. To examine this prediction, we generated monotransrepressors containing the RD of the Krüppel associated box (KRAB) of KOX-1 protein [14] and/or of the mSin3 interaction domain (SID) of Mad1 protein [15] as single copy or multiple copies and examined their abilities to repress transcription from the ERE-bearing promoter constructs driving the expression of a reporter enzyme cDNA. Consistent with our predictions, monotransrepressors effectively decreased the expression of reporter gene in a type- and RD copy number-dependent manner. Importantly, the expression of a monotransrepressor by recombinant adenoviruses in ER-negative MDA-MB-231 cells derived from a breast adenocarcinoma decreased/repressed as well as induced/enhanced gene expressions and cellular growth with polar directions compared to those observed with E2-ERα or monotransactivator. Providing proof-of-principal, our approach could be used for the development of various platforms in genome-wide analysis of gene expressions and epigenetic modifications and also in clinical applications wherein transcription factor-mediated genomic alterations at various loci are predominant.

## Material and Methods

### Engineering of ERE Binding Monotransregulators

The engineering of expression vectors bearing ERα, the ERE binding module CDC and the ERE binding defective counterparts ERα_EBD_ and CDC_EBD_ were described previously [10,12,13,16]. We also described the construction of PV and PV_EBD_ previously [10]. ERα_EBD_ contains amino-acid substitutions (Glu_203_Ala, Gly_204_Ala and Arg_211_Glu) in the DNA recognition helix of the DNA binding domain [10], while CDC_EBD_ contains amino acid substitutions replacing Cys_202_ and Cys_205_ with His residues [12].

**Fig 1:**
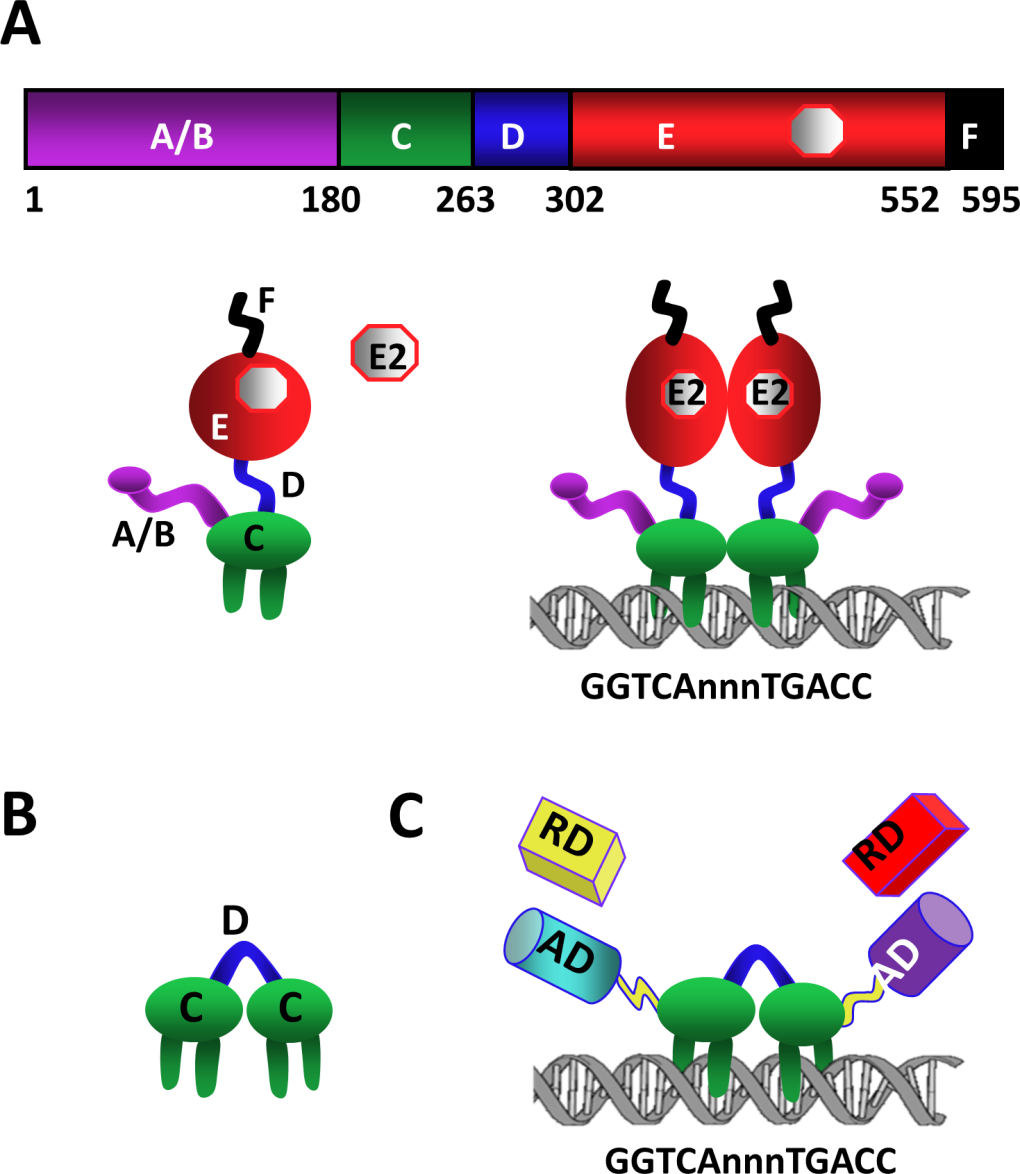
Engineering of monotransregulators. (**A**) Schematic of ERα. 595 amino-acid long ERα is a modular protein that contains an amino-terminally located ligand-independent activation function, A/B, domain followed by the DNA binding, C, domain. The hinge, D, domain encompasses a nuclear localization signal and connects the C domain to the carboxyl-terminus, E/F, domain. E/F is a multi-functional domain containing ligand binding, dimerization and ligand-dependent activation functions. ERα binds to specific DNA sequences, so-called estrogen responsive elements (EREs), derivatives of the consensus GGTCAnnnTGACC wherein ‘n’ denotes three non-specific nucleotides. (**B**) The engineered monomeric ERE binding module CDC is composed of two C domains joined with the D domain. (**C**) The cDNA of the activation domain (AD) of p65 and VP16 proteins or of the repression domain (RD) KRAB of KOX-1 of and/or SID of Mad1 was genetically joined to the 5′ and 3′ ends, respectively, of the CDC-cDNA or the ERE binding defective CDC_EBD_ to generate the monotransregulators. The constructs also contain sequences encoding the Flag and 6xHis epitopes at the amino and carboxyl-termini, respectively.

To generate monotransregulators with repression domains (RDs), we used the RD of KRAB and/or SID cDNA. The cDNA of the KRAB-RD encoding amino acids 1–90 of the KOX-1 protein [14] and the cDNA for SID encoding residues 1–35 of Mad1 protein [15] were generated by PCR using a human testis cDNA library (Clontech Labs Inc., Mountain View, CA, USA). The cDNA for KRAB (K) or SID (S) was then genetically fused as single copy (K-CDC; S-CDC or CDC-K; CDC-S) or multiple copies (KK-CDC; SS-CDC; CDC-KK; CDC-SS) to the 5’ or 3’ of CDC-cDNA to generate monotransregulators (Fig 1C). We also used single copy or multiple copies of K and S cDNAs conjugated to the 5’ or 3’ of the CDC-cDNA (K-CDC-S, S-CDC-K; KK-CDC-SS or SS-CDC-KK). In addition, we generated ERE binding defective counterparts using the cDNA of the CDC_EBD_ by exchanging the CDC cDNA using restriction enzymes. All constructs contain sequences that encode a Flag epitope at the amino-terminus and a 6xHis epitope at the carboxyl-terminus of proteins. Constructs were sequenced for amino-acid fidelity.

For reporter vector, we used pGL3 Luciferase Reporter vector that drives the expression of the *Firefly Luciferase* cDNA as the reporter enzyme. (Promega Corp., Madison, WI, USA). Reporter plasmids bearing single ERE juxtaposed to the 5’ of the Thymidine kinase (TK) or Simian Virus 40 (SV40) promoter were described previously [12]. We also described pGL3 reporter vector bearing the promoter of the *Complement 3* (*C3*) or the *Metalloproteinase 1* (*MMP1*) gene [12,17]. The reporter vector driving the expression of the *Renilla Luciferase* cDNA (Promega Corp.) was also previously described [12,17,18].

Restriction and DNA modifying enzymes were obtained from New England Bio-Labs (Beverly, MA, USA) or Invitrogen (Carlsbad, CA, USA). 17β-estradiol (E2) was purchased from Sigma-Aldrich (St. Louis, MO, USA).

### Cell Cultures and Biochemical Assays

MDA-MB-231 cells were cultured as described [12]. The Flag antibody (M2) without or with horseradish peroxidase (HRP) or fluorescein isothiocyanate (FITC) was purchased from Sigma-Aldrich.

To assess the functional protein synthesis, we used immunocytochemistry (ICC), Western blot (WB) and electrophoretic mobility shift assay (EMSA). Transfected or infected cells were processed for WB, EMSA, and ICC as described previously [9,10,13,17–19]. Briefly, transfected or infected cells for 48h were collected and lysed with three cycles of freeze/thaw in a lysis buffer. Total protein in the amount of 10 μg was subjected to SDS 10%-PAGE and processed for WB. Proteins were probed with the horseradish peroxidase-conjugated monoclonal Flag antibody (M2-HRP, Sigma–Aldrich) and detected with the ECL-Plus Western Blotting kit (GE Healthcare Bio-Sciences, Pittsburgh, PA, USA). The images were captured using PhosphorImager (MolecularDynamics, Sunnyvale, CA, USA). For ICC, infected cells were fixed with 2% paraformaldehyde in PBS and permeabilized using 0.4% Triton X-100 in PBS. Proteins were probed with the Flag M2-FITC (Sigma-Aldrich). DAPI (4,6-diamido-2-phenylindole hydrochloride; Vectashield, Vector Laboratories, Inc., Burlingame, CA) was used for nucleus staining. For EMSA, oligomers bearing single consensus ERE sequence were annealed and ^32^P end-labeled as described [18]. Labeled oligomers were incubated in the absence or presence of equal amounts (10 μg) of cellular extracts without or with the Flag-M2 antibody (1 μg) for the conformation of the specificity of protein-ERE interactions. Reactions were subjected to electrophoresis on a 4% nondenaturing polyacrylamide gel. Images were analyzed with PhosphorImager.

### Generation of recombinant adenoviruses

Recombinant adenovirus bearing none or a cDNA was produced as described previously [9,10,13,20] with the exception that we used the AdenoVator Adenoviral Expression System from Qbiogene Inc. (Carlsbad, CA). The purified viruses were titered using an Adeno-X Rapid Titer Kit (BD Biosciences, Palo Alto, CA, USA) to determine the multiplicity of infection (MOI).

### Endogenous Gene Expression

MDA-MB-231 cells (100,000 cells/well) were plated in 6-well culture plates in phenol red-free medium containing 10% dextran coated charcoal-stripped Fetal Bovine Serum, CD-FBS, without E2 for 24 h. Cells were then infected with Ad5-ERα or Ad5-ERα_EBD_ and maintained without (ethanol, EtOH, %0.01 v/v as vehicle control) or with a physiological concentration, 10^−9^ M, of E2 for 48 h, while Ad5-monotransregulator infected cells were maintained for 48 h in the presence of EtOH. The total RNA was subjected to quantitative PCR (qPCR) using TaqMan probes (Thermo-Fisher Sci. Inc., Foster City, CA, USA) as described [9,10,13]. The relative quantification was performed using the comparative 2^ΔΔC^_T_ method [21].

### Cell Cycle Analysis

#### Flow Cytometry

MDA-MB-231 cells (50,000 cells/well) in 6-well tissue culture plates were infected with recombinant adenoviruses in the absence (EtOH) or presence of 10^−9^ M E2 for 48h. The cells were collected and pelleted. Cells were re-suspended in one ml ethanol (70%) for overnight to fix and permeabilize cells. Cells were subsequently incubated with one mg/ml RNase A (Sigma-Aldrich) for 30 min followed by 20 mg/ml propidium iodide (PI) (Sigma-Aldrich) for 10 min. Cells were then subjected to flow cytometry using EPICS Elite (Coulter Corp, Miami, FL, USA) as described [9,13,22].

#### EdU Incorporation Assay

To directly measure the effects of transregulators on DNA synthesis, we used Click-iT EdU Flow Cytometry Assay Kit (Thermo-Fisher). An alkyne containing EdU (5-ethynyl-2´-deoxyuridine), a nucleoside analog to thymidine, is incorporated into DNA during active DNA synthesis. The incorporated alkyne-EdU is subsequently conjugated to an azide containing Alexa Fluor-647 dye through copper-catalyzed covalent reaction. The EdU incorporation is then measured with flow cytometry. The percentage of EdU-incorporated cells is used to determine the percentage of S-phase cells in the population. Click-IT reaction was carried out as directed by the manufacturer. In brief, MDA-MB-231 cells (50,000 cells/well) in 6-well tissue culture plates were infected with recombinant adenoviruses in the absence (EtOH) or presence of 10^−9^ M E2. 24h after infection, additional fresh medium containing 2.5 μM EdU, without (EtOH) or with 10^−9^ M E2, were added and cells were further maintained for 24h. Cells were then harvested by trypsinization, fixed with Click-IT fixative, permeabilized and subjected to Click-IT reaction in the presence of CuSO_4_ and Fluorescent Dye Azide 647 for 30 min. Cells were washed twice with 1% BSA in PBS and subjected to flow cytometry using 633/635 nm excitation with a red emission filter (660/20 nm).

#### Cellular Proliferation

MDA-MB-231 (20,000 cells/well) cells were plated in 24-well culture plates in phenol red-free medium containing 10% CD-FBS for 24h. Cells were infected with recombinant adenoviruses in the absence (EtOH) or presence of 10^−9^ M E2 for 96h. At termination, cells were subjected to 3-(4,5-Dimethylthiazol-2-yl)-2,5-diphenyltetrazolium bromide (MTT) assay (Invitrogen) [10,13].

### Statistical Analysis

Results were presented as the mean ± standard error (SEM). Significance was determined using a two-tailed unpaired t test with a confidence interval of 95%.

## Results

ERα, as other transcription factors, is a modular protein in that distinct structural regions of the receptor display unique functional features [1,2]. We previously suggested that the intrinsic DNA sequence decoding ability of ERα (Fig 1A) to bind to permutations of ERE can be exploited to generate designer transcription factors by protein engineering [12]. To accomplish this, we engineered an ERE binding module protein, CDC, by joining two DNA binding domains (Cs) of ERα with the hinge domain (D) that contains a nuclear localization signal (Fig 1B). Since ERs share a %96 amino-acid identity in their DBDs, CDC design also affords a generic ERE binder. CDC binds to ERE sequences with an affinity and specificity similar to those observed with the dimer ERα through interactions with the same nucleosides [12]. The integration of single copy or multiple copies of strong AD from VP16 and/or p65, into CDC resulted in monotransactivators that specifically targeted and potently activated ERE-driven heterologous gene expressions independent of dimerization, ligand, ER subtype, promoter and cell context [12]. Moreover, monotransactivators, but not the ERE-binding defective counterparts, altered the expression of endogenous ERE-bearing estrogen responsive genes and cellular proliferation similar to E2-ERα [12,13].

Since the genetic conjugation of ADs to CDC generated monotransactivators, we reason here that the fusion of the repression domain (RD) of the Krüppel associated box (KRAB, K) of KOX-1 and/or of the mSin3 interaction domain (SID, S) of Mad1 protein as single copy or multiple copies to CDC should produce ERE-binding monomeric repressors that effectively suppress the transcription of ERE-bearing genes. To accomplish this, we inserted the PCR-generated cDNAs of K-RD and/or S-RD to the 5′ and/or 3′ ends of CDC-cDNA to generate monotransrepressors (Fig 1C). To ensure that the effects of transregulators on gene expressions and subsequent cellular changes are due to the ability of constructs to interact with EREs, we also generated ERE binding defective monotransrepressors using the ERE-binding defective CDC_EBD_ as the template. The monotransactivator PV bearing the ADs of p65 (P) and VP16 (V) proteins at the amino- and carboxyl-termini respectively, and the ERE binding defective counterpart, PV_EBD_, were described previously [12]. All constructs contain sequences encoding Flag and 6xHis epitopes at the amino- and carboxyl-termini, respectively, for protein detection.

**Fig 2:**
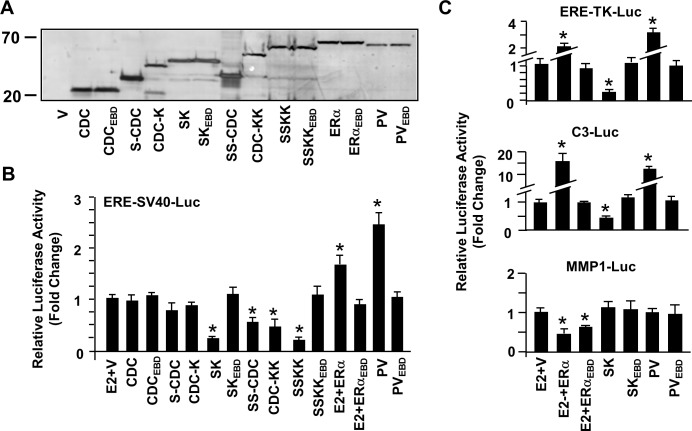
Synthesis and transcriptional activity of monotransregulators in transfected cells. **(A)** MDA-MB-231 cells were transiently transfected with 62.5 ng pcDNA expression vector bearing none (V) or a construct cDNA. Cell extracts (10 μg) 48h after transfections were subjected to WB using the horse radish peroxidase (HRP)-FIag M2 antibody. Molecular masses in kDa are shown. **(B & C)** Cells were transfected with 62.5 ng pcDNA expression vector bearing none (V) or a construct cDNA together with the *Firefly Luciferase* (Luc) reporter plasmid in the amount of 125 ng. The reporter plasmid contained one consensus ERE located upstream of the Simian virus 40 (SV40) or the Thymidine kinase (TK) promoter; or the native promoter sequences derived from the estrogen responsive *Complement 3* (*C3*) or *Metalloproteinase 1* (*MMP1*) gene. The transfection efficiency was monitored by the co-expression of 0.5 ng of a reporter plasmid, pCMV-RL that drives the expression of *Renilla Luciferase* cDNA. Cells were treated without (EtOH) or with 10^−9^ M E2 (for Vector, ERα and ERα_EBD_) and without E2 for transregulators for 24h. The cell extracts were assayed for reporter enzymes, and the normalized *Firefly/Renilla Luciferase* activities are presented as fold changes compared to the parent vector in the absence of E2, which was set to one. Shown are the mean ± SEM of three independent experiments performed in duplicate.

To initially study the synthesis of constructs, we transiently transfected ER-negative MDA-MB-231 cells, derived from a breast adenocarcinoma, with a mammalian expression vector bearing none (V) or a cDNA for a monotransrepressor, monotransactivator, ERα or an ERE-binding defective counterpart. Results revealed that transfected cells synthesize proteins at expected molecular masses determined by WB analysis using the HRP-Flag-M2 antibody (Fig 2A).

To examine the transactivity of constructs, we transiently transfected MDA-MB-231 cells with a reporter plasmid bearing none (SV40-Luc) or single consensus ERE juxtaposed to the 5’-end of the strong SV40 promoter that drives the expression of the *Firefly Luciferase* enzyme cDNA as the reporter, ERE-SV40-Luc, together with an expression vector bearing none (V) or a construct cDNA (Fig 2B). Cells were also co-transfected with a *Renilla Luciferase* reporter vector for the determination of transfection efficiency. Normalized luciferase values revealed that CDC, CDC with single SID at the amino-terminus (S-CDC) or with single KRAB at the carboxyl-terminus (CDC-K) had minimal effects on reporter enzyme activity. On the other hand, the construct bearing S or K as multiple copies (SS-CDC or CDC-KK) repressed the reporter enzyme activity. This suggests that an increase in the number of the same species of RD enhances the repression potency of monotransrepressors. Importantly, CDC bearing both S and K as single copy (S-CDC-K) or multiple copies (SS-CDC-KK) was more effective in repressing the enzyme activity compared to constructs bearing the same species as multiple copies at one terminus (SS-CDC or CDC-KK) or at both termini (SS-CDC-SS or KK-CDC-KK, data not shown). The enhanced repressive capacity of S-CDC-K, which was similar to those observed with SS-CDC-KK, is likely due to the recruitment of distinct, in addition to the common, regulatory complexes by RDs to the promoter. In clear contrast, ERα in response to 10^−9^ M E2 or PV enhanced the reporter enzyme activity. As expected, none of the ERE binding defective counterparts (CDC_EBD_, S-CDC_EBD_-K, SS-CDC_EBD_-KK, PV_EBD_ or ERα_EBD_) affected reporter enzyme levels. It should be noted that the constructs had no effect on reporter from the SV40 promoter without an ERE (SV40-Luc, data not shown). Based on repression levels, we selected S-CDC-K, SK, as the model monotransrepressor to be used in subsequent experiments.

We also observed similar effects (Fig 2C) of constructs on *Firefly Luciferase* levels from the moderately strong thymidine kinase promoter (TK) with single consensus ERE juxtaposed to the 5’-end of the promoter (ERE-TK-Luc) but not from TK without ERE (data not shown). While E2-ERα and PV enhanced, SK effectively repressed reporter enzyme levels. Likewise, SK suppressed whereas PV and E2-ERα augmented the *Luciferase* enzyme level from the reporter plasmid bearing the promoter region of the third component of complement 3 (*C3*), a substrate for the C3-cleaving enzymes of the complement cascade, gene that confers responsiveness to E2-ERα through an ERE [5,23] (Fig 2C). The DNA-binding defective counterparts had no effect on enzyme levels.

SK, as PV, lacks the ERα-specific transactivation domains critical for the regulation of gene expression through the ERE-independent signaling route [24]. We therefore anticipated that SK would be ineffective in modulating gene expression from a reporter plasmid that simulates the ERE-independent signaling pathway in experimental systems. To test this prediction, we used, as we described previously [12], the immediate promoter of the human Metalloproteinase 1, *MMP1*, (MMP1-Luc) gene which responds to E2-ERα through functional interactions with the activator protein 1 (AP1) bound to an AP1 site on the promoter [24]. We found that ERα and ERα_EBD_ in response to 10^−9^ M E2 repressed the enzyme activity (Fig 2C). On the other hand, SK, PV or ERE binding defective counterparts had no effect on reporter enzyme levels.

Our results indicate that SK effectively represses, whereas E2-ERα and PV enhance, transcription from heterologous reporter systems that emulate the ERE-dependent signaling pathway.

Although the ‘activator’ or ‘repressor’ functions were expectedly reflected in the reporter systems used here, we previously showed that PV, as E2-ERα, increases as well as decreases the expression of endogenous estrogen responsive genes in cells [13]. This suggests that the chromatin context of a responsive gene promoter is critical for the directionality of gene expression. To assess and to comparatively examine the ability of SK to regulate the expression of endogenous genes, we used recombinant adenoviruses (Ad5) to effectively deliver cDNAs into MDA-MB-231 cells. The amount (multiplicity of infection, MOI) of Ad5 bearing ERα (100 MOI), PV (200 MOI), ERα_EBD_ (150 MOI) and PV_EBD_ (900 MOI) cDNAs was based on our previous studies in which infected MDA-MB-231 cells synthesize comparable level of proteins [13]. The concentration of Ad5-SK and Ad5-SK_EBD_, which was 100 and 500 MOI, respectively, was derived from preliminary studies (S1 Fig). Recombinant adenoviruses at indicated MOIs produced transregulator proteins at levels comparable to that of ERα at 48h post-infection in MDA-MB-231 cells. For experiments, the total MOI was adjusted to 900 MOI, which was the highest concentration of recombinant adenovirus bearing the PV_EBD_ cDNA, by supplementing with the parent adenovirus (Ad5).

**Fig 3:**
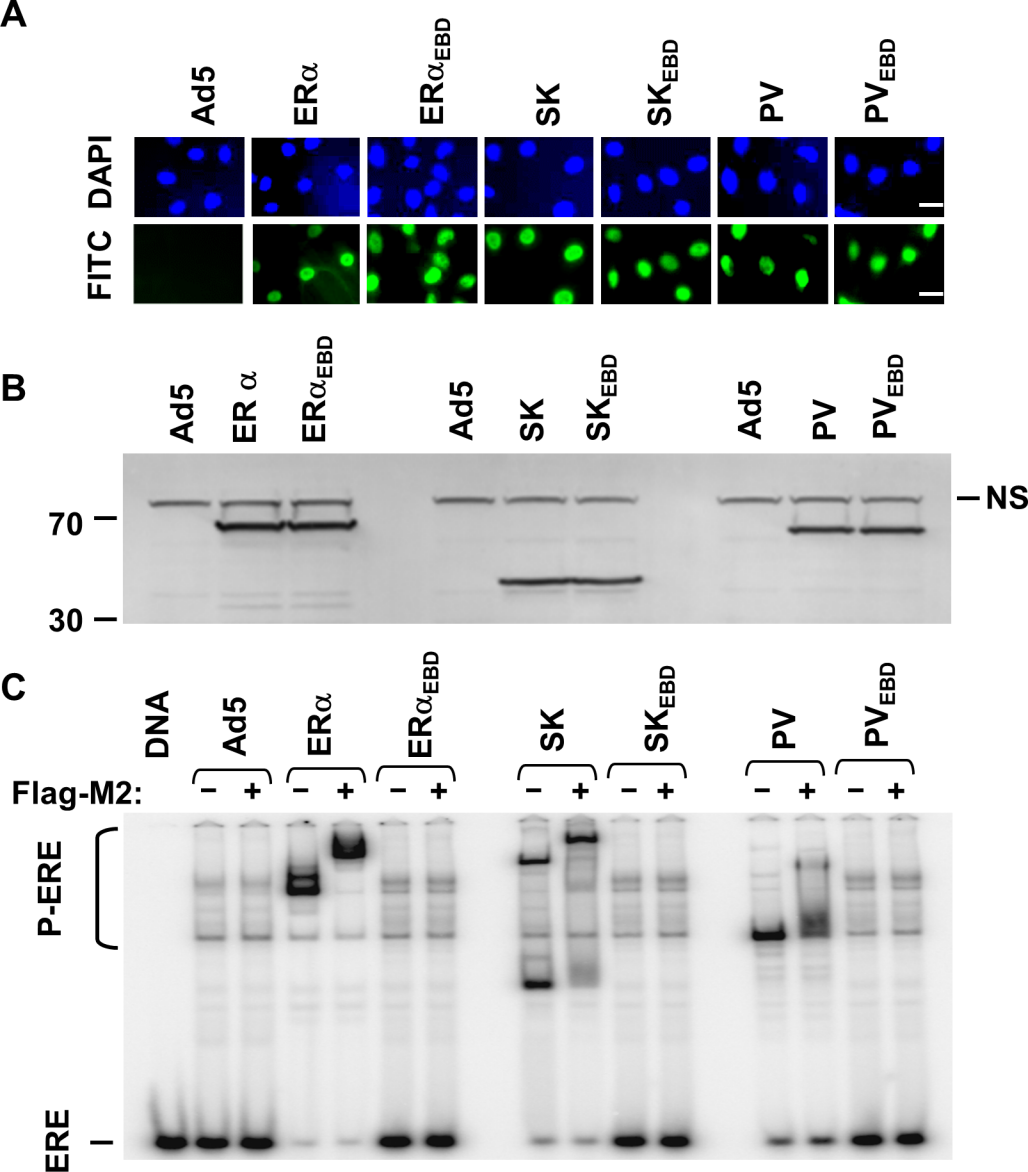
Intracellular localization, synthesis and ERE interaction of transregulators in MD-MB-231 cells. (**A**) Cells were infected with recombinant adenovirus bearing none (Ad5) or a cDNA. At 48 h post-infection, the fluorescein isothiocyanate (FITC)-conjugated Flag-M2 antibody localizes proteins in the nucleus stained with 4′,6-diamidino-2-phenylindole (DAPI). Scale bar is 20 μm. (**B**) Cell extracts (10 μg) at 48 h post-infection were subjected to WB using the HRP conjugated-FIag M2 antibody. NS denotes a non-specific protein band. Molecular masses in kDa are shown. (**C**) Cell extracts (10 μg) at 48h post-infection were also subjected to electrophoretic mobility shift assay (EMSA) with (+) or without the Flag-M2 antibody (Flag-M2). ERE specifies the unbound and P-ERE denotes the protein-bound radiolabeled ERE. DNA indicates the radiolabeled ERE only. In all experiments, a representative result from three independent determinations is shown.

As ERα, PV and the ERE binding defective counterparts, SK and SK_EBD_ are localized in the nucleus assessed by immunocytochemistry (ICC) in infected MDA-MB-231 cells (Fig 3A). WB analysis using the HRP-Flag-M2 antibody indicated that infected cells synthesize monotransregulators with the expected molecular masses (Fig 3B). Electrophoretic mobility shift assay (EMSA) showed that SK, like ERα and PV, but not ERE binding defective counterparts, interacts with ERE (Fig 3C). That the Flag-M2 antibody (+) further retarded the electrophoretic migration of proteins bound to the radiolabeled ERE indicates the specific protein-ERE interactions.

### Effects of Monotransregulators on Gene Expression

**Fig 4:**
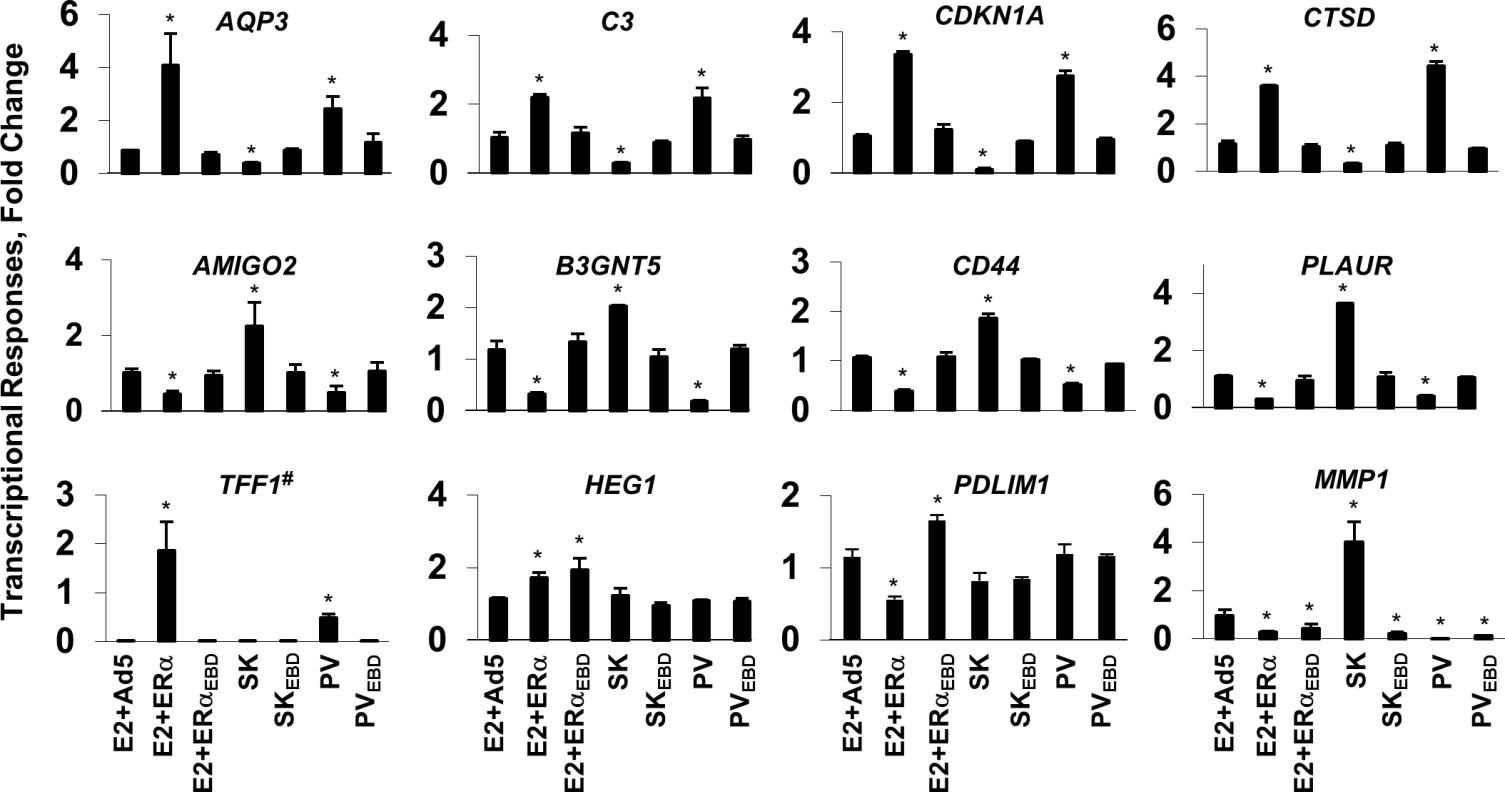
Regulation of endogenous gene expressions by monotransregulators. MDA-MB-231 cells infected with the adenovirus bearing none (Ad5), ERα or ERα_EBD_ cDNA were treated without (data not shown) or with 10^−9^ M E2 for 48 h, while cells infected with the adenovirus bearing monotransregulator cDNA were treated with EtOH vehicle. Total RNA was subjected to qPCR for the analysis of estrogen responsive gene expressions. Results, which are the mean ± SEM of three independent determinations in triplicates, depict fold changes in mRNA levels compared with those observed in cells infected with Ad5 in the absence of E2, which is set to 1. Asterisk (*) indicates significant change. Note that TFF1^#^ denotes fold changes in hundreds.

To examine the effects of monotransregulators on gene expressions, we selected a subset of estrogen responsive genes regulated through ERE-dependent or ERE-independent pathways, which we previously determined using a microarray approach [9,10]. To accomplish this, we infected MDA-MB-231 cells with recombinant adenoviruses for 48h in the absence (EtOH) or presence of 10^−9^ M E2 for Ad5, ERα and ERα_EBD_ or in the absence of E2 (EtOH, 0.01%) for monotransregulators and subjected them to total RNA extraction and qPCR. We found that E2-ERα and PV induced whereas SK but not SK_EBD_ repressed the expression of the *AQP3* (Aquaporin 3), *C3* (Complement component 3), *CDKN1A* (Cyclin dependent kinase inhibitor, p21) and *CTSD* (Cathepsin D) genes (Fig 4) as well as the *B4GALT1* (UDP-Gal:betaGlcNAc beta 1,4- galactosyltransferase, polypeptide 1), *HK1* (Hexokinase 1), *MANEAL* (Mannosidase, Endo-Alpha-Like) and *TBXA2R* (thromboxane A2 receptor) genes (data not shown). On the other hand, SK, but not SK_EBD_, enhanced, in contrast to E2-ERα and PV which repressed, the expression of the *AMIGO2* (adhesion molecule with Ig-like domain 2), *B3GNT5* (UDP-GlcNAc:betaGal beta-1,3-N-acetylglucosaminyltransferase 3), *CD44* (CD44 Antigen), *PLAUR* (plasminogen activator, urokinase receptor) genes (Fig 4) together with the *GCNT2* [glucosaminyl (N-acetyl) transferase 2, I-branching enzyme (I blood group], *HS3ST3B1* [heparan sulfate (glucosamine) 3-O-sulfotransferase 3B1], *TGFB2* (transforming growth factor, beta 2) and *UAP1* (UDP-N-acteylglucosamine pyrophosphorylase 1) genes (data not shown). These findings suggest that the regulation of this subset of genes by SK, like E2-ERα and PV, requires ERE interactions and indicate that a transregulator with activator or repressor domains can mediate the expression of the same gene with polar directions within a chromatin context.

*TIFF1* (trefoil factor 1, pS2) *RARA* (retinoic acid receptor, alpha) or *TGFA* (transforming growth factor, alpha) expression is regulated by E2-ERα through the ERE-dependent signaling pathway [10,13]. E2-ERα or PV, but not the ERE binding defective counterpart, indeed effectively induced the expression of the *TFF1* (trefoil factor 1) (Fig 4), *RARA* (retinoic acid receptor, alpha) and *TGFA* (transforming growth factor, alpha) genes (data not shown). On the other hand, SK had no effect on the expression of these genes. Responsiveness of these genes to PV or E2-ERα but not to SK suggests a quiescent state of the cognate promoter that is poised only for activation.

The *HEG1* (heart development protein with EGF-like domains 1) or *PDLM1* (PDZ and LIM domain 1) gene expression mediated by E2-ERα is an example for the ERE-independent signaling pathway [10]. *HEG1* and *PDLM1* expressions were indeed responsive only to E2-ERα and E2-ERα_EBD_ (Fig 4). These results are consistent with our expectations that PV or SK lacking ER-specific regulatory domains would be ineffective in regulating the expression of ERE-independent gene network.

We previously showed that E2-ERα and PV as well as the ERE-binding defective counterparts modulate the expression of the *MMP1* (Matrix metallopeptidase 1) or *HAS2* (Hyaluronan synthase 2) genes [13], implying the generality of action. Similarly, all constructs, regulated the *MMP1* (Fig 4), *MMP3* (Matrix metallopeptidase 3), *SERPINB2* [Serpin peptidase inhibitor, clade B (ovalbumin), member 2] or *HAS2* (Hyaluronan synthase 2) gene expression (data not shown). Interestingly, SK enhanced, the others repressed, the expression of *MMP1*, suggesting a distinct mechanism in gene regulation.

Constructs had no effect on the transcription of the *CCND1* (Cyclin D1), *JAK1* (Janus kinase 1) or *IRS1* (insulin receptor substrate 1) gene, which was used as control (data not shown).

### Mediation of Cellular Growth

**Fig 5:**
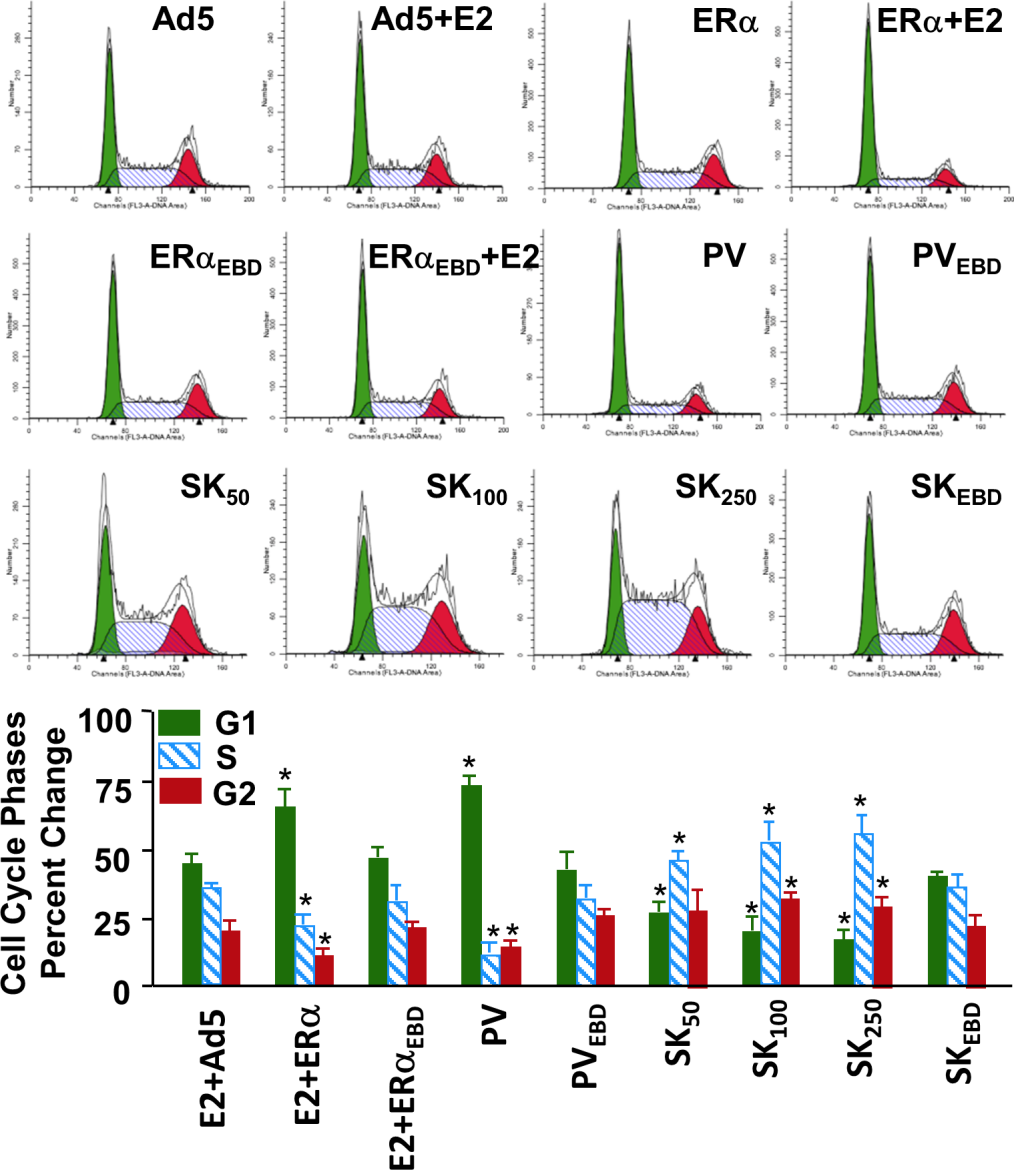
Effects of monotransregulators on cell cycle phases. MDA-MB-231 cells infected with recombinant adenoviruses in the absence (EtOH) or presence (E2) of 10^−9^ M E2 for 48h were subjected to flow cytometry for cell cycle analysis. Results, with representative histograms, are depicted as the percentage of cells in G1, G2 and S phases and are the mean ± SEM of three independent experiments.

**Fig 6:**
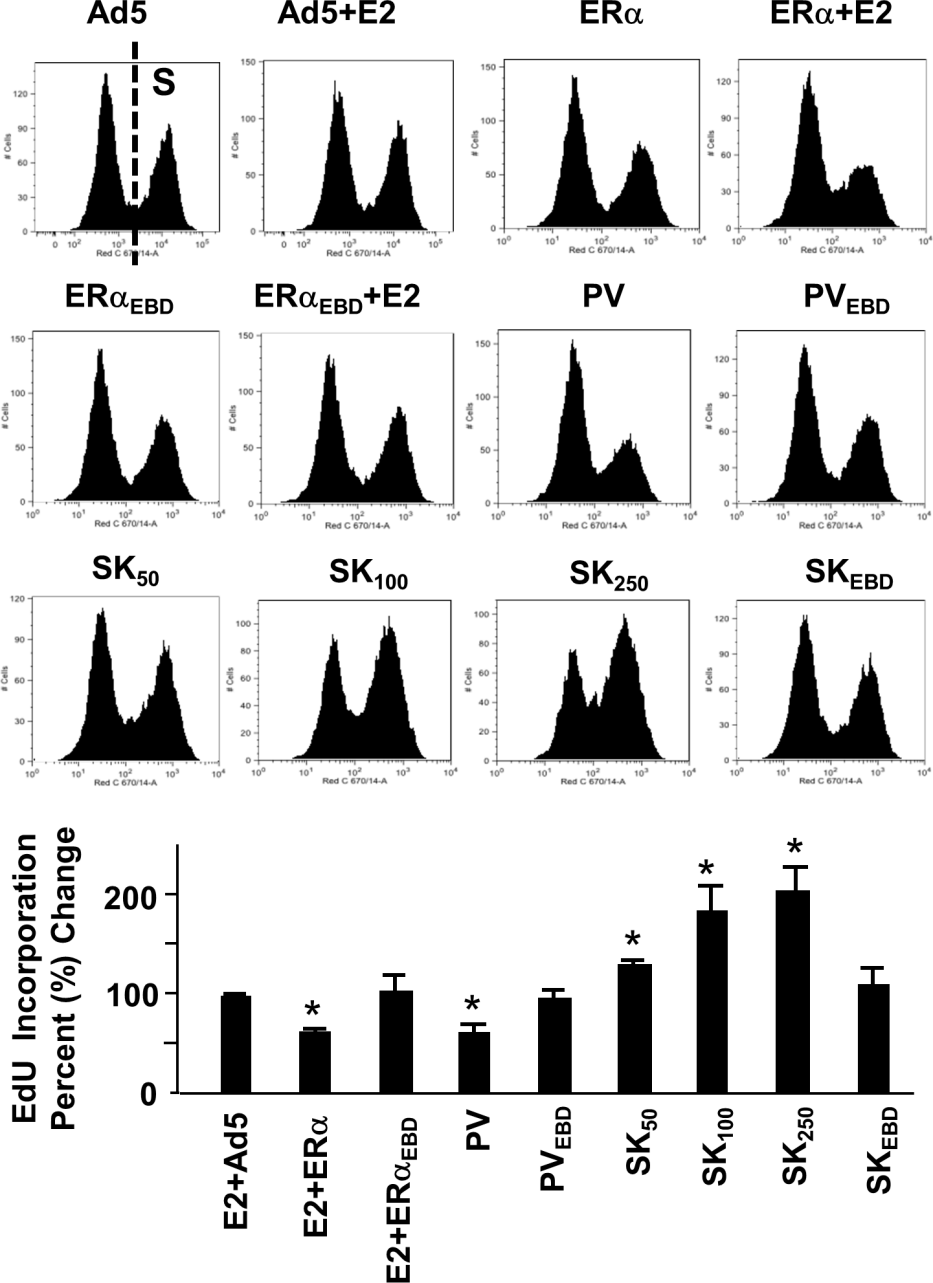
Effects of monotransregulators on DNA synthesis. MDA-MB-231 cells infected with recombinant adenoviruses in the presence (E2) or absence (EtOH) of 10^−9^ M E2 for 24h. Cells were then treated with EdU, without (EtOH) or with 10^−9^ M E2, and further maintained for 24h. Cells were then subjected to Click-IT EdU DNA content assay and flow cytometry. Results, with representative histograms indicating proliferating cells (S), which incorporated EdU, are depicted as the percent change in EdU incorporation in cells compared to those infected with Ad5 and are the mean ± SEM of three independent experiments. Asterisk (*) depicts significant change.

We previously showed that genomic responses from the ERE-dependent signaling pathway to E2-ERα are necessary for the alterations in cellular features [9,13] and that PV alters cellular proliferation in a manner similar to E2–ERα [12,13]. Since SK modulates the expression of ERE-bearing estrogen responsive genes with polar directions compared to those regulated by E2-ERα or PV, we wanted to examine whether SK also induces changes in cellular proliferation in the opposite direction. To accomplish this, we infected MDA-MB-231 cells with recombinant adenoviruses for 48h in the absence (EtOH) or presence of 10^−9^ M E2. Cells were then subjected to cell cycle analysis. Results revealed, as we showed previously [10,13], that E2-ERα and PV, but not ERE-binding defective mutants, increased the number of cells accumulated in G1 phase while decreasing it in S and G2 phases compared to those observed with the parent Ad5 (Fig 5). In clear contrast, SK at all concentrations tested, but not SK_EBD_ at any concentration (shown at 500 MOI), increased the cell number accumulated in S and G2 phases with a corresponding decrease in G1 phase (Fig 5). These results suggest that SK in contrast to PV or E2-ERα augments DNA synthesis. To examine this prediction, infected MDA-MB-231 cells were subjected to the EdU incorporation assay. This assay is based on the incorporation of alkyne containing EdU (5-ethynyl-2´-deoxyuridine), a nucleoside analog of thymidine, into DNA during the active DNA synthesis. The percentage of EdU-incorporated cells determined by flow cytometry indicates that SK indeed increases while PV or E2-ERα decreases DNA synthesis (Fig 6). The absence of any effect of ERE-binding defective mutant proteins on EdU incorporation into DNA further emphasizes the importance of protein-ERE interactions in the regulation of DNA synthesis and cell cycle.

**Fig 7:**
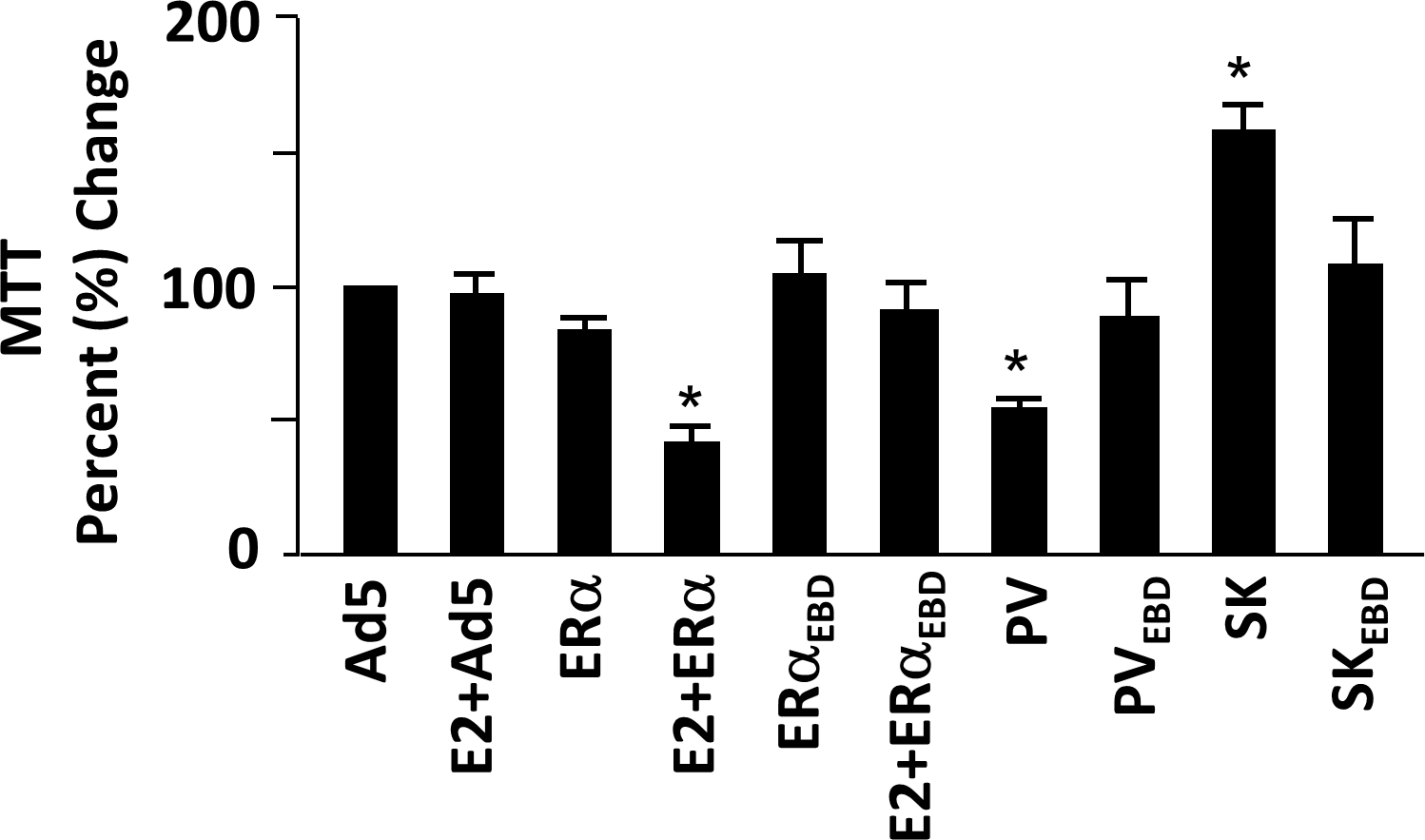
Effects of monotransregulators on cellular growth assessed by MTT assay. Infected MDA-MB-231 cells were maintained in the absence (EtOH) or presence of 10^−9^ M E2 (E2) for 96h were subjected to 3-(4,5-dimethylthiazol-2-yl)-2,5-diphenyltetrazolium bromide (MTT) assay. The mean ± SEM, which depicts three independent experiments performed in duplicate, indicates percentage (%) change in color as an indication of cellular proliferation compared to that observed in cells infected with Ad5 in the absence of E2, which is set to 100. Asterisk (*) denotes significant change.

Alterations in DNA synthesis as well as in the distribution of cell cycle phases were reflected in cellular growth assessed by an MTT assay of infected MDA-MB-231 cells at 96h (Fig 7). We found that SK increased, whereas PV or E2-ERα repressed, the cellular proliferation, while the ERE binding defective counterparts were without an effect on cell growth.

## Discussion

Our results collectively show that the integration of potent RDs from other transcription factors into the engineered monomeric ERE-binding module generates a transregulator SK that not only represses but also activates ERE-bearing genes with polar directions to those observed with E2-ERα and PV, the latter which contains ADs. The monotransrepressor SK also modulated DNA synthesis, cell cycle progression and proliferation in opposite directions compared to those mediated by E2-ERα and PV. Our results indicate that a transcription ‘activator’ or a ‘repressor’ is bi-potential within a chromatin context in that it possesses both transcription activating/enhancing and repressing/decreasing abilities.

The control of gene expressions is a dynamic process and is ultimately determined by the local chromatin alterations initiated by transcription factors as nucleation units for various chromatin modifiers. The concept of transcription activator or repressor has been used for transcription factors that activate or repress gene expression, respectively [25]. Transcriptional activators are, for example, proteins that assemble the transcriptional machinery at the promoter of a gene for the induction of transcription. The assembly of transcription machinery is carried out by ADs of a transcription factor that interacts combinatorially and sequentially with a variety of co-regulatory complexes and the basal transcription machinery [26–28]. The strong AD of the herpes simplex virion protein, VP16 [29,30], or the p65 subunit of the nuclear factor κB, NFκB, [31] has been widely used in the engineering of artificial transcription factors (ATFs) designed to bind to a pre-determined DNA sequence and to activate transcription from reporter systems and/or endogeneous promoters [32–34]. Similarly, the genetic conjugation of RDs to ATFs generates potent transcriptional repressors. The KRAB domain of Krüppel zinc-finger proteins displays DNA-binding dependent suppression of transcription that requires the ubiquitously expressed KAP-1 as the co-repressor [35–37]. The KAP-1 in turn recruits nucleosome remodeling histone deacetylase complex (NuRD), histone deacetylases (HDACs), histone methyltransferases and heterochromatin protein 1, leading to gene silencing [37]. In addition, specific inhibition of some components of RNA polymerase II by KRAB is also involved in transcription suppression [36,38].

Transcriptional repression by the basic helix-loop-helix zipper protein Mad1 requires its heterodimer partner Max and the co-repressor protein Sin3. The interaction between Mad1 and Sin3 is mediated by SID [15,39,40]. SID recruits Nuclear Receptor Co-Repressors (NCoR/SMRT) and HDACs to form a repressive complex for transcription [15,39,40]. Thus, the repression of gene expression mediated by KRAB or SID using the same as well as distinct co-regulatory proteins is ultimately linked to local chromatin modifications that are non-permissive for transcription.

Early studies indicated that the KRAB or SID domain suppresses transcription when tethered to DNA: The fusion of KRAB to the DNA binding domain of LexA or Gal4 transcription factor, for example, effectively silences the transcription of reporter genes in transfected cells [35]. Similarly, we observed here that monotransrepressors, including SK, decreased the expression of reporter genes in heterologous expression systems. On the other hand, SK activated as well repressed endogenous gene expressions. We and others have shown that the expression of the *AQP3*, *AMIGO*, *C3*, *B3GNT5*, *CD44*, *CDKN1A*, *CTSD*, *PLAUR*, *RARA*, *TGFA* and *TIFF1* genes are mediated by E2-ERα through the ERE-dependent signaling pathway [10,13,23,41–50]. We found that SK repressed whereas PV, like E2-ERα, enhanced *AQP3*, *C3*, *CDKN1A* and *CTSD* gene expressions. Conversely, SK induced while PV, and E2-ERα, suppressed the expression of the *AMIGO*, *B3GNT5*, *CD44* and *PLAUR* genes. Polar directions in gene expressions mediated by SK and PV were also reflected in cellular growth in that SK augmented whereas PV, as E2-ERα, suppressed DNA synthesis and cellular proliferation. The ERE-binding defective counterparts, on the other hand, had no effect on the expression of these genes and subsequent cellular responses. Pointing to common mechanisms in the regulation of gene expressions at the ERE-dependent signaling pathway, the mimicry of E2-ERα functions by PV suggests that the chromatin context of an estrogen responsive gene promoter determines the direction of gene expression. Since, however, SK induces the same gene expressions with directions opposite to PV, our findings also emphasize the remarkable flexibility of chromatin modifications that are primarily governed by the nature of a transcription factor. This, as suggested [51,52], implies that the pre-assigned function for an engineered transcription factor as an activator or a repressor at a chromatin context may not be accurate and could be specific to target gene or cell type. Indeed, genome-wide studies, reviewed by Reynolds et al., [25], clearly indicate that many transcriptional repressors are also connected with actively transcribed regions; on the other hand, many activators are, in addition to active regions, associated with repressed loci that show cellular variability.

We also observed that SK did not affect the expression of *TIFF1*, *RARA* or *TGFA* gene in clear contrast to PV or E2-ERα. The inability of SK to alter the expression of some of the estrogen responsive genes mediated through the ERE-dependent signaling pathway might be due to an already repressed state of transcription in chromatin context that cannot be further suppressed but is poised for induction in response to a stimulus as observed with PV or E2-ERα.

The underlying mechanisms of transcriptional modulation of estrogen responsive genes through the ERE-independent pathway are unclear. Studies suggest that functional E2-ERα interactions through ADs at both termini with transcription factors bound to their cognate regulatory elements are critical for the ability of the complex to modulate responsive gene expressions in a ligand-, promoter- and cell-context–dependent manner [24,53]. We previously also suggested that E2-ERα mediates the expression of *HEG1 MMP1*, *MMP3*, *PDLM1 or SERPINB2* gene through the ERE-independent pathway [10]. The inabilities of SK and PV or ERE binding defective counterparts to regulate the transcription of *HEG1* and *PDLM1* which were responsive to only E2-ERα and the DNA binding defective E2-ERα_EBD,_ which modulates the expression with the same (*HEG1*) or opposite direction (*PDLM1*) to that of E2-ERα, are consistent with the importance of receptor-specific features on the expression of these genes. On the other hand, all constructs modulated the expression of *MMP1*, *MMP3* or *SERPINB2* gene. Although implying a generality of action, the ability of SK to modulate the expression of, for example, *MMP1* and that of E2-ERα_EBD_ to regulate *PDLM1*, also suggest a distinct mode of transcriptional regulation.

Regardless of mechanisms, it is apparent that the regulation of cellular proliferation in opposite directions by SK with RDs and PV with ADs as bi-potential transcription factors is dependent upon estrogen responsive genes mediated by the ERE-dependent signaling pathway. Targeted gene expressions and/or genome modifications have critical importance in biological research and in clinical setting. Zinc Finger Proteins (ZFPs), Transcription Activator-Like Effectors (TALEs) and Clustered Regulatory Interspaced Short Palindromic Repeat/Cas9 based RNA-guided DNA endonucleases (CRISPR/Cas9) are the basis of powerful engineering technologies with a wide usage for targeted modulation of gene expressions as well as genome and epigenome manipulations [54–57]. They are also valuable for gene therapy wherein enhancing or attenuating single gene function could have therapeutic effects. Estrogen tissue malignancies, on the other hand, are multigenic wherein genome-wide regulation of large number of responsive genes by ER antagonists signifies clinical benefits [2,58]. Consequently, targeted transcriptional modulation of gene network by transregulators for desired phenotypic effect could be a promising approach and yet necessitates testing it in various cellular contexts. Our approach, nevertheless, provides proof-of-principle and could be useful for the better understanding of the roles of ERE-driven gene network in the manifestation of phenotypic characteristics of cell models that emulate different breast neoplasms and also for the development of novel therapeutic reagents.

## Supporting Information

S1 Fig Synthesis and interaction with ERE of monotransregulators in MDA-MB-231 cells. (**A**) Synthesis of monotransregulators as a result of different concentrations of recombinant adenoviruses. Cells were infected with recombinant adenoviruses bearing none (Ad5) or a cDNA at indicated MOIs. In all infections, the total MOI was adjusted to 1500, which is the highest concentration of adenovirus used in the experimental series, by supplementing with Ad5. Extracts (10 μg) of infected cells at 48h were subjected to WB using the HRP-Flag M2 antibody. Molecular mases in kDa is indicated. NS denotes non-specific protein detection. (**B**) Cell extracts (10 μg) at 48h post-infection were also subjected to electrophoretic mobility shift assay (EMSA) without (-) or with (+) the Flag-M2 antibody (Flag-M2). ERE specifies the unbound and P-ERE denotes the protein-bound radiolabeled ERE. DNA indicates the radiolabeled ERE lane only.
(TIF)
