# Supplementary figures and images for: Modulation of Estrogen Response Element-Driven Gene Expressions and Cellular Proliferation with Polar Directions by Designer Transcription Regulators
Source: PLoS One. 2015 Aug 21;10(8):e0136423. doi: 10.1371/journal.pone.0136423 (PMC4546503; doi:10.1371/journal.pone.0136423)

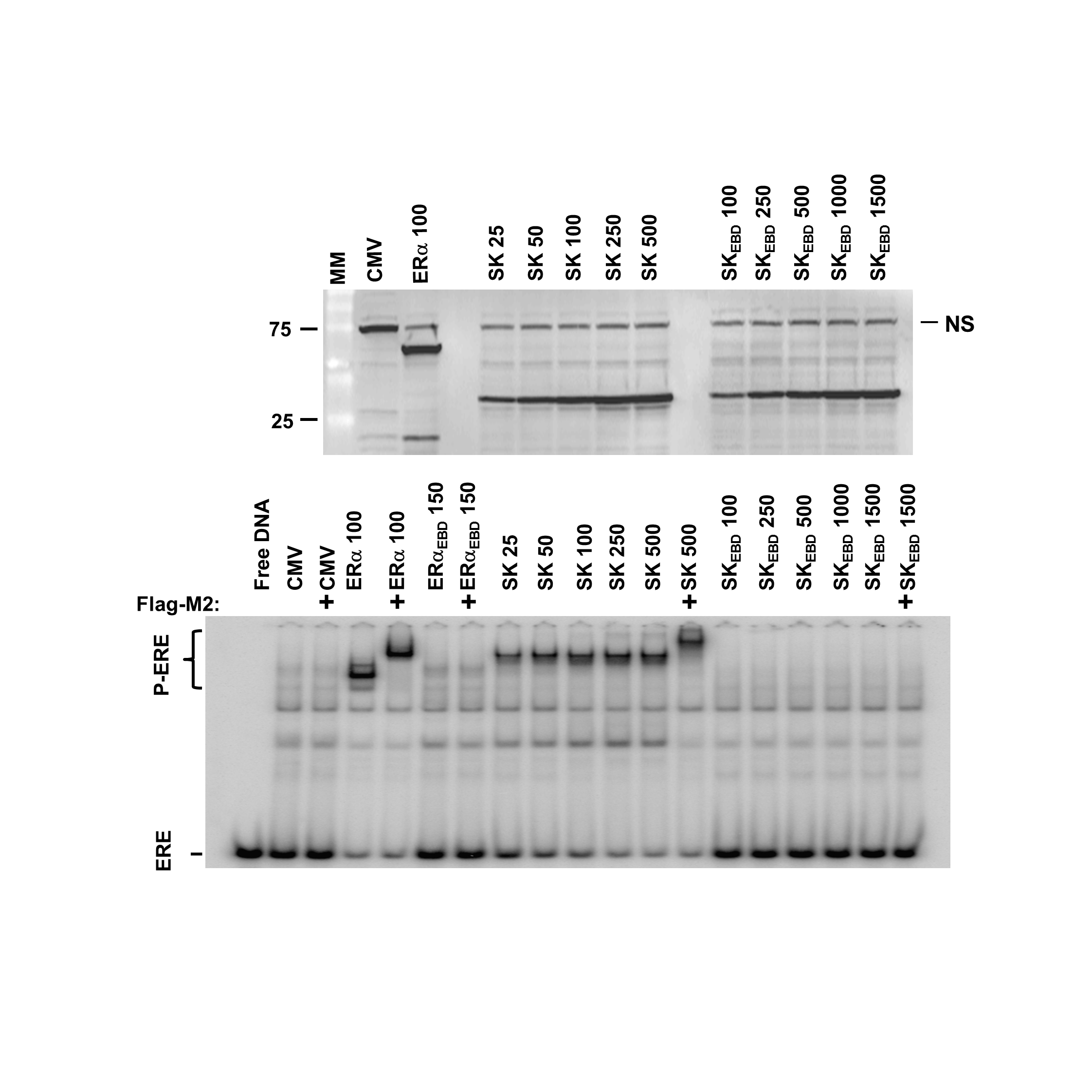

Supplement: S1 Fig — (A) Synthesis of monotransregulators as a result of different concentrations of recombinant adenoviruses. Cells were infected with recombinant adenoviruses bearing none (Ad5) or a cDNA at indicated MOIs. In all infections, the total MOI was adjusted to 1500, which is the highest concentration of adenovirus used in the experimental series, by supplementing with Ad5. Extracts (10 μg) of infected cells at 48h were subjected to WB using the HRP-Flag M2 antibody. Molecular mases in kDa is indicated. NS denotes non-specific protein detection. (B) Cell extracts (10 μg) at 48h post-infection were also subjected to electrophoretic mobility shift assay (EMSA) without (-) or with (+) the Flag-M2 antibody (Flag-M2). ERE specifies the unbound and P-ERE denotes the protein-bound radiolabeled ERE. DNA indicates the radiolabeled ERE lane only. (TIF) [file pone.0136423.s001.TIF]
